# Safety in treatment of hepatocellular carcinoma with immune checkpoint inhibitors as compared to melanoma and non-small cell lung cancer

**DOI:** 10.1186/s40425-017-0298-2

**Published:** 2017-11-21

**Authors:** Zachary J. Brown, Bernd Heinrich, Seth M. Steinberg, Su Jong Yu, Tim F. Greten

**Affiliations:** 1 Thoracic and Gastrointestinal Oncology Branch, Center for Cancer Research, National Cancer Institute, National Institutes of Health, Bethesda, MD 20892 USA; 2 Biostatistics and Data Management Section, Center for Cancer Research, National Cancer Institute, National Institutes of Health, Bethesda, MD 20892 USA

**Keywords:** Hepatocellular carcinoma, Immunotherapy, Immune checkpoint inhibitors, Adverse events

## Abstract

**Background:**

Hepatocellular carcinoma (HCC) is a major health problem worldwide with increasing incidence rates. As HCC traditionally occurs in chronically inflamed livers, this inflammation aids to drive oncogenesis and often renders these lesions to be immunogenic and therefore potential targets for immunotherapy. As patients with HCC generally have underlying liver dysfunction, we sought to determine if immune checkpoint inhibitors were safe to use in patients with HCC as compared to melanoma and non-small cell lung cancer (NSCLC) in terms of the gastrointestinal side effects of elevation of aspartate aminotransferase (AST), alanine aminotransferase (ALT), and diarrhea as well as patients who drop out of the study due to drug toxicity and death secondary to drug toxicity.

**Methods:**

A literature review was performed for clinical trials that have been completed with single agent immune checkpoint inhibitors for patients with HCC, melanoma, and NSCLC. Gastrointestinal related adverse events including elevation of aspartate aminotransferase (AST), alanine aminotransferase (ALT), and diarrhea were analyzed as well as those patients who were taken off therapy secondary to drug related toxicity and patients who died as a result of therapy.

**Results:**

We found that although patients with HCC treated with immune checkpoint inhibitors have a substantial increase in AST/ALT as compared to patients with melanoma and NSCLC, this does not cause the patients to come off therapy or cause death secondary to drug toxicity.

**Conclusions:**

We propose immune checkpoint inhibitors are safe to pursue in the treatment of HCC.

## Background

Hepatocellular carcinoma (HCC) is a major health problem worldwide. Globally, HCC is the second leading cause of cancer related death [1]. As per the annual report to the Nation on the Status of Cancer, deaths from liver cancer increased at the highest rate of all cancer sites, and liver cancer incidence rates increased sharply [2]. For patients with advanced diseased, sorafenib is the standard of care showing a survival advantage from 7.9 to 10.7 months as compared to placebo [3]. Recently, the RESORCE trial demonstrated an additional survival advantage of approximately 3 months in patients treated with regorafenib following progression on sorafenib, which led to approval for regorafenib in HCC by the US Food and Drug Administration (FDA) [4]. Thus, there is a high need for new treatment options to improve patient survival.

Immunotherapy is a fast-moving field that has shown promise in other malignancies, notably melanoma, but is quickly evolving as a treatment for HCC [5–8]. Immunotherapy appears to be a suitable treatment option for HCC as HCC traditionally occurs in chronically inflamed livers, such as those infected with hepatitis B or C and patients with non-alcoholic fatty liver disease. This inflammation aids to drive oncogenesis and often renders these lesions to be immunogenic [9–11]. As a result, the tumors often express tumor-associated antigens and neo-antigens that arise from specific gene mutations which make attractive targets for the immune system [8]. However, due to a variety of stromal cells and immunoinhibitory molecules, these antitumor immune responses are often blunted with immune inhibitory checkpoints having been recognized as having an increasing role in tumor escape [8, 12].

Immune checkpoint inhibitors such as the anti-CTLA-4 antibody Ipilimumab or the anti-PD-1 antibody Nivolumab or Pembrolizumab have been FDA-approved for treatment of melanoma and non-small cell lung cancer (NSCLC). Recently, several clinical trials showed promising results for the use of immune checkpoint inhibitors in HCC [13–15]. Unlike traditional chemotherapy, immunotherapy acts indirectly through modulating the immune system in an attempt to promote immune recognition for a lasting antitumor response [8]. However, like traditional chemotherapy, immunotherapy including checkpoint inhibitors are not without adverse events. A unique gamut of adverse events from checkpoint inhibitors may occur due to activation of the immune system and induction of autoimmunity [16–18]. Such adverse events involve the gastrointestinal system including hepatitis, transaminitis, diarrhea and colitis. Other organ systems are also affected as well including but not limited to pneumonitis, hypophysitis, as well as endocrine dysfunction and skin reactions such as pruritus and rash [16, 19]. Immune-related adverse events after checkpoint blockade in general have been reviewed elsewhere [20, 21]. Here we would like to focus on HCC patients, considering their impaired liver function due to underlying liver disease and tumor burden.

The first immune checkpoint inhibitor to enter clinic trials was ipilimumab, an CTLA-4 blocking antibody, whose approval by the FDA was based on the results from trials in patients with advanced melanoma with an acceptable adverse event profile [19, 22–25]. However compared to melanoma patients, patients with HCC are more likely to have liver dysfunction as a result of chronic viral hepatitis and fibrosis as well as tumor burden in the liver. The first clinical trial in HCC with immune checkpoint inhibitors was by Sangro et al. looking at tumor response in patients with HCC and chronic hepatitis C. When designing the trial, the authors expressed their concern for the risk of inducing immune-mediated fulminant hepatitis because of the checkpoint blockade [14]. Historically, patients with viral hepatitis have been excluded from prior clinical trials with checkpoint blockade. Duffy et al. administered Tremelimumab to HCC patients and combined their treatment with radiologic ablation therapies [13]. Recently, El-Khoueiry et al. reported results of a phase I/II study of nivolumab in HCC which consisted of dose escalation and expansion phases and is the first reported trial treating HCC with a checkpoint inhibitor targeting PD-1 [15]. The above mentioned trials had acceptable safety profiles and showed potential for future treatment of HCC.

In this study, we performed a literature review and summary of data from clinical trials with checkpoint inhibitors in HCC, melanoma, and NSCLC. Although patients with hepatocellular carcinoma generally have preexisting underlying liver dysfunction as the result of hepatitis or cirrhosis, we found patients with HCC treated with checkpoint inhibitors have a higher incidence of transaminitis but there is no difference in the percentage of patients taken off the immune checkpoint inhibitor secondary to drug toxicity or death secondary to drug toxicity.

## Methods

We performed a literature review of clinical trials that have been performed with single agent immune checkpoint inhibitors for patients with HCC, melanoma, and NSCLC on PubMed. Immune checkpoint inhibitors included in this study targeted CTLA-4 and PD-1 which comprised of tremelimumab, nivolumab, ipilimumab and pembrolizumab. Agents targeting PD-L1 were excluded as only very limited information in abstract form is available about studies testing anti-PD-L1 in HCC. We analyzed study arms with treatment only consisting of single agent immune checkpoint inhibitors. Study arms containing combination therapy with chemotherapy or other immunotherapy agents were excluded.

We analyzed gastrointestinal related adverse events including elevation of aspartate aminotransferase (AST), alanine aminotransferase (ALT), and diarrhea based upon grading criteria (ie National Cancer Institute Common Terminology Criteria for Adverse Events) categorized as grade 1–5 toxicities as reported per clinical trial. We included episodes of colitis in the diarrhea group as some studies did not distinguish between the two adverse events. Additionally, although trials in melanoma and NSCLC report drug induced hepatitis as an adverse event, this was excluded from our analysis as most patients in the HCC studies had underlying chronic hepatitis at baseline as compared to patients with melanoma and NSCLC and was not reported as an adverse event in those trials. Other indicators of hepatic dysfunction, such as elevation of total bilirubin, low albumin and international normalized ratio (INR), were not utilized as they were generally not reported in the clinical trial adverse events. We also analyzed those patients who were taken off therapy secondary to drug related toxicity as well as those patients who died as a result of therapy.

For each of the studies, the percentage of patients with each type of adverse event was determined by dividing the total number of patients with that event by the number of patients in the study. The percentages were then compared among all three groups (HCC, NSCLC, and melanoma) using an exact form of a Kruskal-Wallis test. Then, the percentages were individually compared between the studies reporting on HCC and each of the other two diagnoses using an exact form of a Wilcoxon rank sum test. The *p*-values are two-tailed and presented without formal adjustment for multiple comparisons. However, in view of the number of types of adverse events evaluated, for the three-group comparison, *p* < 0.01 would indicate a significant overall difference among disease categories, while 0.01 < *p* < 0.05 would indicate a trend. For the comparison of HCC with NSCLC, there are only three trials vs. 5–6 being compared, and with this number of studies, the smallest theoretical *p*-value that can be obtained by an exact Wilcoxon rank sum test is 0.036; thus, this indicates a difference for these groups, but limited ability to consider truly the comparisons as being statistically significant. For comparison of HCC with melanoma, *p* < 0.01 would indicate a significant difference, while 0.01 < *p* < 0.05 would indicate a trend.

## Results

Data from three studies reporting on adverse events in HCC, six studies reporting adverse events in NSCLC and 16 studies reporting adverse events in patients with melanoma were analyzed after literature review excluding studies that did not contain a study arm containing single agent immune checkpoint inhibitors or reported adverse events secondary to drug toxicity (Table 1). Clinical trials in HCC with immune checkpoint inhibitors include one trial using tremelimumab alone, one trial combining tremelimumab with tumor ablative therapies and one trial with nivolumab in a dose expansion phase for a total of 314 patients. For NSCLC, we identified six trials, three with pembrolizumab and three with nivolumab for a total of 1866 patients. For clinical trials with immune checkpoint inhibitors in melanoma we identified 16 trials, five with ipilimumab, three with tremelimumab, three with pembrolizumab, and five with nivolumab for a total of 4118 patients (Table 2).

**Table 1:** Clinical trials with immune checkpoint inhibitors and given adverse event profiles

Drug	Author	Tumor Type	Number of Patients	Taken off therapy secondary to toxicity	Death secondary to therapy	Elevation AST any grade	Elevation AST grade 3–4	Elevation ALT any grade	Elevation ALT grade 3–4	Diarrhea any grade	Diarrhea grade 3–4
Tremelimumab	Sangro et al. 2013 [14]	HCC	20	3	0	14	9	11	5	6	1
Tremelimumab + ablation	Duffy et al. 2016 [13]	HCC	32	4	0	11	7	6	3	2	0
Nivolumab	El-Khoueiry et al. 2017 [15]	HCC	262	9	0	26	14	24	8	32	3
Pembrolizumab	Garon et al. 2015 [33]	NSCLC	495	NR	NR	15	3	11	2	40	3
Pembrolizumab	Reck et al. 2016 [34]	NSCLC	154	11	1	NR	NR	NR	NR	22	6
Pembrolizumab	Herbst et al. 2016 [35]	NSCLC	682	32	6	17	2	24	3	52	6
Nivolumab	Borghaei et al. 2015 [36]	NSCLC	287	14	1	9	1	9	0	22	2
Nivolumab	Brahmer et al. 2015 [37]	NSCLC	131	4	0	2	0	2	0	10	0
Nivolumab	Rizvi et al. 2015 [38]	NSCLC	117	14	2	0	0	1	0	12	3
Ipilimumab	Wolchok et al. 2010 [39]	Melanoma	214	35	0	NR	NR	NR	NR	66	14
Ipilimumab	Hodi et al. 2010 [23]	Melanoma	131	17	4	1	0	2	0	46	13
Ipilimumab	Robert et al. 2015 [40]	Melanoma	256	24	1	6	2	9	2	79	26
Ipilimumab	Eggermont et al. 2015 [22]	Melanoma	471	245	5	78	20	102	25	270	81
Ipilimumab	Larkin et al. 2015 [41]	Melanoma	311	46	1	12	5	11	2	139	46
Tremelimumab	Camacho et al. 2009 [42]	Melanoma	89	10	0	NR	NR	NR	NR	34	13
Tremelimumab	Ribas et al. 2013 [43]	Melanoma	325	43	7	NR	NR	NR	NR	166	60
Tremelimumab	Kirkwood et al. 2010 [44]	Melanoma	246	13	2	NR	NR	NR	NR	99	28
Pembrolizumab	Robert et al. 2014 [45]	Melanoma	173	6	0	5	0	6	0	22	1
Pembrolizumab	Robert et al. 2015 [40]	Melanoma	555	30	0	20	1	16	1	102	21
Pembrolizumab	Ribas et al. 2015 [46]	Melanoma	357	17	0	NR	NR	NR	NR	34	2
Nivolumab	Topalian et al. 2014 [47]	Melanoma	107	17	0	4	0	5	0	19	2
Nivolumab	Robert et al. 2015 [48]	Melanoma	210	14	0	2	1	3	2	33	2
Nivolumab	Weber et al. 2015 [49]	Melanoma	268	7	0	11	1	7	2	30	1
Nivolumab	Larkin et al. 2015 [41]	Melanoma	313	24	1	12	3	12	4	64	9
Nivolumab	Weber et al. 2016 [50]	Melanoma	92	0	0	5	0	6	0	39	1

*HCC* Hepatocellular Carcinoma, *NSCLC* Non-small cell lung cancer, *AST* aspartate aminotransferase, *ALT* alanine aminotransferase, *NR* not reported

**Table 2:** Total adverse events reported per cancer

Disease	Number of Patients	Taken off therapy secondary to toxicity	Death secondary to toxicity	Elevation AST any grade	Elevation AST grade 3–4	Elevation ALT any grade	Elevation ALT grade 3–4	Diarrhea any grade	Diarrhea grade 3–4
HCC	314	16	0	51	30	41	16	40	4
NSCLC	1866	75	10	43	6	47	5	161	22
Melanoma	4118	548	21	156	33	179	38	1242	320

*HCC* Hepatocellular Carcinoma, *NSCLC* Non-small cell lung cancer, *AST* aspartate aminotransferase, *ALT* alanine aminotransferase

**Fig. 1:**
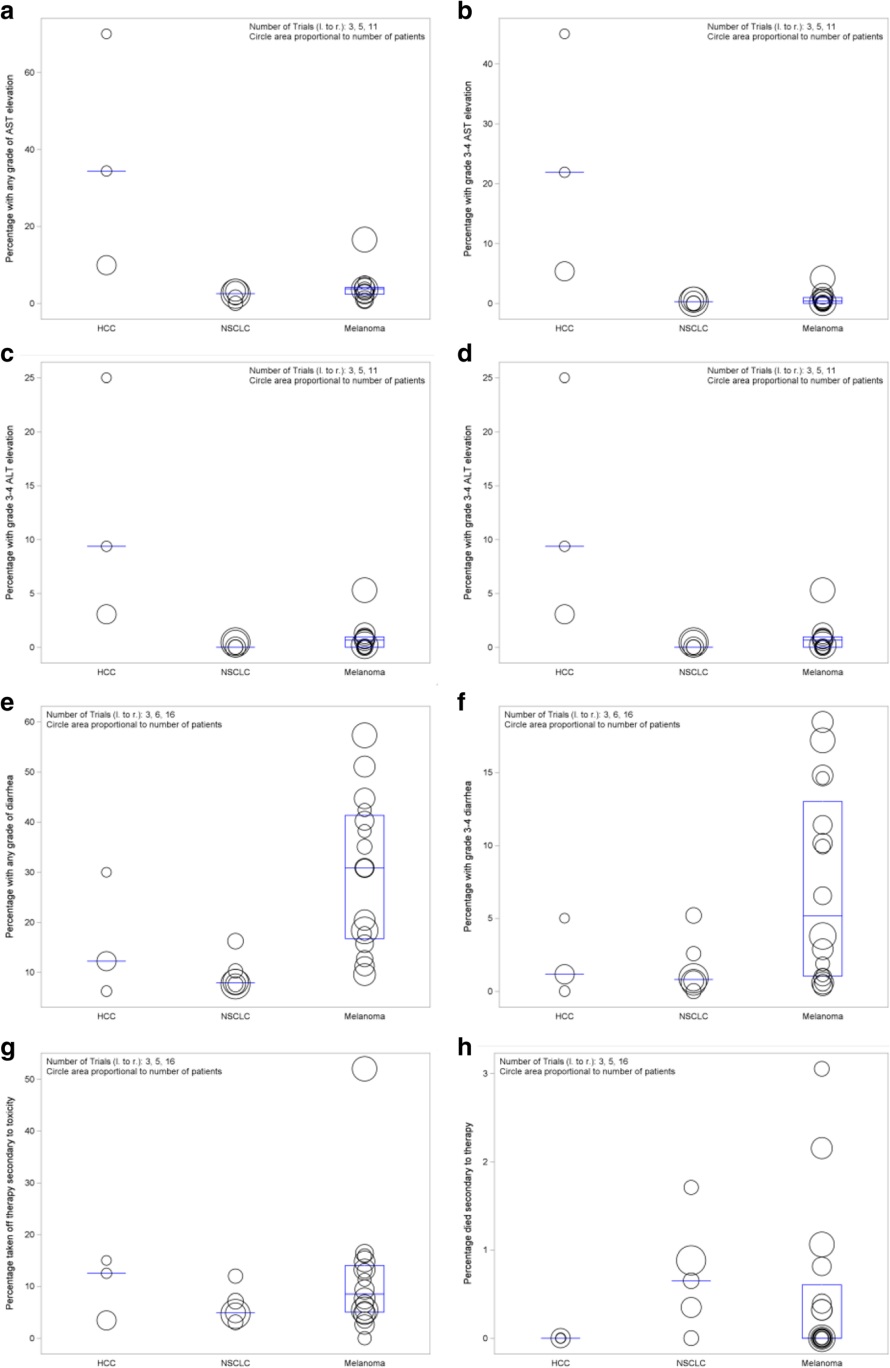
Percentage of patients with adverse events in checkpoint inhitor clinical trials. Circles represent individual clinical trials and size of the circles represent the number of patients enrolled in the study (larger the circle equals greater number of patients). **a**: AST elevation of any grade. **b**: AST elevation grade 3–4. **c**: ALT elevation of any grade. **d**: ALT elevation grade 3–4. **e**: Diarrhea of any grade. **f**: Diarrhea grade 3–4. **g**: Patients taken off therapy secondary to drug toxicity. **h**: Patients died secondary to therapy. HCC: Hepatocellular Carcinoma, NSCLC: Non-small cell lung cancer, AST: aspartate aminotransferase, ALT: alanine aminotransferase

For overall comparison of the three groups of trials, elevation of AST or ALT of any grade differed significantly among patients with the three types of disease (*p* = 0.0051 and *p* = 0.0083 respectively), as did grade 3–4 AST or ALT toxicity (*p* = 0.0096 and *p* = 0.0067 respectively; Table 3, Fig. 1a, b, c, and d). Diarrhea of any grade also differed significantly among patients in the three disease groups (*p* = 0.00079) but there was no statistical difference in grade 3–4 diarrhea (*p* = 0.12) among the groups (Table 3, Fig. 1e and f). There was no difference among the three groups with respect to patients discontinuing therapy secondary to drug toxicity (*p* = 0.48) or deaths secondary to drug toxicity (*p* = 0.12; Table 3, Fig. 1g and h).

**Table 3:** Sample statistics on proportions of patients in N trials with adverse events as shown. *P*-values are by an exact form of the Kruskal-Wallis test for comparison of the trial results among all three disease types, while they are by an exact form of the Wilcoxon rank sum test for comparison of trial results between HCC and NSCLC or melanoma

								Overall Comparison	HCC vs NSCLC	HCC vs melanoma
Disease	Variable	Mean	N	Standard Error	Lower Quartile	Median	Upper Quartile	*p*-value	*p*-value	*p*-value
HCC	Taken off therapy secondary to toxicity	0.10	3	0.04	0.03	0.13	0.15	0.48	0.39	0.96
	Death secondary to therapy	0.00	3	0.00	0.00	0.00	0.00	0.12	0.11	0.34
	Elevation AST any grade	0.38	3	0.17	0.10	0.34	0.70	0.0051	0.036	0.011
	Elevation AST grade 3–4	0.24	3	0.12	0.05	0.22	0.45	0.0096	0.036	0.0028
	Elevation ALT any grade	0.28	3	0.14	0.09	0.19	0.55	0.0083	0.036	0.022
	Elevation ALT grade 3–4	0.12	3	0.07	0.03	0.09	0.25	0.0067	0.036	0.0055
	Diarrhea any grade	0.16	3	0.07	0.06	0.12	0.30	0.00079	0.71	0.11
	Diarrhea grade 3–4	0.02	3	0.02	0.00	0.01	0.05	0.12	0.96	0.25
NSCLC	Taken off therapy secondary to toxicity	0.06	5	0.02	0.05	0.05	0.07			
	Death secondary to therapy	0.01	5	0.00	0.00	0.01	0.01			
	Elevation AST any grade	0.02	5	0.01	0.02	0.02	0.03			
	Elevation AST grade 3–4	0.00	5	0.00	0.00	0.00	0.00			
	Elevation ALT any grade	0.02	5	0.00	0.02	0.02	0.03			
	Elevation ALT grade 3–4	0.00	5	0.00	0.00	0.00	0.00			
	Diarrhea any grade	0.10	6	0.01	0.08	0.08	0.01			
	Diarrhea grade 3–4	0.02	6	0.01	0.01	0.01	0.03			
Melanoma	Taken off therapy secondary to toxicity	0.11	16	0.03	0.05	0.09	0.14			
	Death secondary to therapy	0.01	16	0.00	0.00	0.00	0.01			
	Elevation AST any grade	0.04	11	0.01	0.02	0.04	0.04			
	Elevation AST grade 3–4	0.01	11	0.00	0.00	0.00	0.01			
	Elevation ALT any grade	0.05	11	0.02	0.03	0.04	0.05			
	Elevation ALT grade 3–4	0.01	11	0.00	0.00	0.01	0.01			
	Diarrhea any grade	0.30	16	0.04	0.17	0.31	0.41			
	Diarrhea grade 3–4	0.07	16	0.02	0.01	0.05	0.13

In subgroup analyses comparing studies of patients with HCC and NSCLC, there were greater proportions of HCC patients exhibiting elevations in AST and ALT of any grade (both *p* = 0.036) as well as grade 3–4 AST or ALT elevation (both *p* = 0.036). There was no difference in the rate of diarrhea between groups of patients for any grade toxicity (*p* = 0.71) and grade 3–4 toxicity (*p* = 0.96). Additionally, there was no statistically significant difference in the proportions of patients with dose limiting toxicity causing those patients to come off the study (*p* = 0.39) as well as death secondary to toxicity (*p* = 0.11, Table 3, Fig. 1).

Comparing HCC and melanoma studies, there was a trend toward significantly greater proportions of HCC patients with any-grade toxicity with respect to AST and ALT elevation (*p* = 0.011 and *p* = 0.022 respectively). Significantly higher proportions of HCC patients also exhibited grade 3–4 elevation of AST and ALT (*p* = 0.0028 and *p* = 0.0055 respectively). There was no statistical difference between patients with HCC or melanoma with respect to diarrhea of any grade (*p* = 0.11) or with grade 3–4 toxicity (*p* = 0.25). Again, there was no statistical significance in drug limiting toxicity causing patients to come off the study (*p* = 0.96) as well as death secondary to toxicity (*p* = 0.34 Table 3, Fig. 1).

## Discussion

Sorafenib is the current standard of care in advanced and end-stage HCC with the SHARP trial showing a meager survival advantage of 3 months compared to placebo [3]. A recent trial with regorafenib following progression of sorafenib showed an additional survival benefit of approximately 3 months [4]. It has been considered no small feat to have a positive phase III clinical trial as progress toward successful therapies has been slow [26]. HCC is an immunogenic tumor where better overall survival and time to recurrence has been predicted by the presence of tumor infiltrating lymphocytes [27]. However, as compared to other patient populations such as those patients with melanoma or non-small cell lung cancer, patients with HCC generally have chronic underlying liver disease caused by viral hepatitis or more recently non-alcoholic steatohepatitis. It has been shown that immune checkpoint inhibitors can lead to liver dysfunction with hepatitis and elevation of AST and ALT [16]. Immune checkpoint inhibitors have been utilized in recent HCC clinical trials with promising results. Similarly, checkpoint inhibitors can lead to pneumonitis and patients with non-small cell lung cancer have favorable results without terrible toxicity. Therefore, it is reasonable to ask whether it is safe to use immune checkpoint inhibitors in patients with hepatocellular carcinoma with likely baseline hepatic dysfunction even though these drugs can cause GI side effects of elevation of AST and ALT as well as diarrhea and colitis.

As patients with viral hepatitis have been excluded from clinical trials of checkpoint inhibitors in other disease states, the effect on patients infected with hepatitis B or C was largely unknown. Additionally, pre-clinical data in mouse models is largely missing in this cohort of patients as the hepatitis viruses cannot infect mice. Therefore, as mouse models do exist to mimic viral hepatitis, it was unknown whether administration of a checkpoint inhibitor may cause hepatocyte destruction due to an overwhelming immune response against infected hepatocytes [28].

Sangro et al. published the first study of using immune checkpoint inhibitors in HCC using tremelimumab in patients with concurrent chronic infection with hepatitis C virus (HCV). Twenty patients were enrolled all of whom had inoperable HCC along with chronic HCV infection and received prior treatment with sorafenib, systemic chemotherapy, or participated in another clinical trial. All patients were required to have an Eastern Cooperative Oncology Group (ECOG) performance status of 0 or 1 as well as adequate tests of organ function such as AST/ALT less than 5 times the upper limit or normal and Child-Pugh A or B. The investigators found an acceptable toxicity profile as well as a mean time to progression of 6.48 months and median overall survival of 8.2 months. In addition, treatment with tremelimumab induced a decrease in HCV viral load [14].

Similarly, Duffy et al. combined tremelimumab with tumor ablation with the hypothesis that tumor ablation would release antigens making the tumor a better immunologic target. Tumor ablation in the form of radiofrequency ablation or cryoablation was performed 36 days after initiation of tremelimumab. Thirty-two patients were enrolled, 19 patients with HCV and five patients with hepatitis B (HBV). Again, they found an acceptable safety profile with median time to tumor progression of 7.4 months and median overall survival of 12.3 months. Twelve of 14 patients with a measurable HCV viral load experienced reduction in their viral load. In the patients with HBV, no viral reactivation was encountered and hepatitis B surface antigen was found to decrease [13].

El-Khoueiry et al. recently published results of the first trial in HCC with nivolumab [15]. In the dose escalation phase, the overall objective response rate was 15% with three complete responses and four partial responses with a median overall survival of 15 months. Of the 48 patients in the dose escalation phase, one patient discontinued treatment due to treatment related increase in AST and ALT but did not have change in liver function. Nivolumab 3 mg/kg was chosen in the dose expansion phase of the study with a reported objective response rate of 20% with three complete responses and 39 partial responses. The median time to progression was 4.1 months. Although the study was not powered to compare patients who were infected with HCV or HBV and those uninfected, subgroup analysis of the dose expansion phase observed responses regardless of infection status. Unlike the previous two studies with tremelimumab, nivolumab exhibited limited antiviral activity. As with the studies described above, there was an acceptable safety profile among patients treated with the checkpoint inhibitors with nine of 262 patients discontinuing treatment due to treatment related drug toxicity. Of note, in the three studies discussed the most common reason for treatment discontinuation was disease progression.

One of the main limitations of this analysis revolves around patient selection in the study of HCC. In general, patients selected for clinical trials in HCC have an acceptable performance status and relatively well-preserved liver function. As was discussed in the evaluation of the SHARP trial and more recently the RESORCE trial, patients selected had well-preserved liver function and performance status and therefore the results and safety profiles must come into question with the real-world application to patients who do not fit these characteristics [3, 4, 26]. The definitive etiology of transaminitis is difficult to define in a HCC study population as the elevation in AST or ALT may be related to a number of factors including tumor progression or drug related toxicity. Additionally, as part of the analysis we could not divide data into transaminitis before and after treatment. For example, the studies of immune checkpoint inhibitors in HCC allow patients to enroll in the study if AST/ALT is less than five times the upper limit of normal. However, based on this analysis, these patients may be categorized into grade 1 or 2 elevation before they receive therapy. Another limitation of this study is the limited number of trials performed in HCC, and thus patients enrolled with immune checkpoint inhibitors in HCC are low compared to melanoma.

## Conclusion

Immunotherapy is gaining increasing interest in treating a wide variety of cancers as well as other diseases such as infectious processes [29]. Currently there are 11 open clinical trials registered at ClinicalTrials.gov involving hepatocellular carcinoma and pembrolizumab, two involving tremelimumab and ten with nivolumab including a phase 3 study of nivolumab vs sorafenib as first line treatment [30]. In addition, durvalumab, a monoclonal antibody blocking PD-L1, is currently being tested in HCC as a single agent or in combination with tremelimumab [31, 32]. This study demonstrates immune checkpoint inhibitors are safe to use in the treatment of HCC. Among patients treated with immune checkpoint inhibitors, a significantly greater proportion of patients with HCC develop an increase in AST and ALT as compared to patients with melanoma and NSCLC. However, the patients with HCC generally have underlying liver dysfunction before treatment is initiated and this elevation in AST and ALT, indicating possible underlying liver dysfunction, is not sufficiently problematic to cause patients to drop out of the clinical trial and stop therapy. Therefore, it is reasonable to continue to pursue treatment of HCC with immune checkpoint inhibitors and potentially use combination therapy of these medications keeping in mind hepatotoxicity of combination therapy has yet to be evaluated in HCC and will require vigilant monitoring in clinical trials.

## Abbreviations

ALT: Alanine Aminotransferase
AST: Aspartate Aminotransferase
CTLA-4: Cytotoxic T-lymphocyte-associated protein-4
ECOG: Eastern Cooperative Oncology Group
HBV: Hepatitis B virus
HCC: Hepatocellular carcinoma
HCV: Hepatitis C virus
NR: Not reported
NSCLC: Non-small cell lung cancer
PD-1: Programmed cell death-1
